# Molecular Mechanism of Cyanidin-3-*O*-Glucoside Disassembling Aβ Fibril In Silico

**DOI:** 10.3390/nu15010109

**Published:** 2022-12-26

**Authors:** Jihui Gao, Jiahui Fu, Xiaoyu Gao, Dong Yang

**Affiliations:** Beijing Key Laboratory of Functional Food from Plant Resources, College of Food Science & Nutritional Engineering, China Agricultural University, 17 East Tsinghua Rd., Beijing 100083, China

**Keywords:** Aβ, fibril, disassembly, cyanidin-3-*O*-glucoside, Alzheimer’s disease

## Abstract

The deposition of β-amyloid (Aβ) in the brain leads to neurotoxic effects and subsequent Alzheimer’s disease (AD). While AD is becoming more and more prevalent in modern society, therapeutic efforts targeting Aβ could be a promising solution. Currently, two natural products are reported to disintegrate preformed Aβ fibril in vitro. Meanwhile, the chemical driving force behind this phenomenon remains unknown. Taking cyanidin-3-O-glucoside (Cy-3G) as an example, here we studied its interaction with different Aβ polymorphs in silico. Negative charges on different Aβ polymorphs draw the interaction with the flavylium cation on Cy-3G. Our results show that Aβ in a single peptide form in solution exposed more hydrophobic solvent accessible surface area than its fibril structure (per protomer), and Cy-3G interacts more intensively with the single peptide form than fibril as indicated by more hydrogen bonding formed and more amino acid residues involved in their hydrophobic interactions. Thus, the single Aβ peptide aggregation into fibril and fibril dissociation into single peptide equilibrium could be disturbed by the preferential binding of Cy-3G to the monomeric Aβ peptide, which leads to the disassembly of the pathogenic Aβ fibril. This study offers a novel perspective of Cy-3G alleviated AD syndrome beyond its dogmatic antioxidant activity.

## 1. Introduction

Alzheimer’s disease, the most prevalent aging-related dementia, has become increasingly common in modern society [1]. The deposition of Aβ in the brain and subsequent neuron and synaptic loss has been the character of AD for almost 30 years [2]. Unfortunately, therapies targeting Aβ have not proved their efficacy in preventing AD progression and have raised many doubts [3,4]. However, the recent approval of aducanumab possibly restrengthens the validity of curing AD via targeting Aβ [5]. With emerging targets against AD, it may evolve into a multifactorial disease with multiple therapeutic sites [6,7]. Interestingly, naphthoquinone and anthraquinone derivatives could target different sites in AD with similar structure scaffolds [8].

Two major types of Aβ peptides are involved in AD, Aβ_40_ and Aβ_42_, and the amino acid sequence of the latter is that of the former with two more residues at the C-terminus [9]. Although the total concentration of Aβ_40_ is higher than that of Aβ_42_, the latter is the major peptide species in parenchymal plaques [10,11,12]. Additionally, the Aβ_40_ fibril prepared in vitro adopts different morphologies, including straight fibrils and twisted morphology [13,14,15]. The irrelevancy of the Aβ_40_-related pathology and the ambiguity of its structural morphology has rendered this peptide to be studied less than others. On the other hand, oligomeric Aβ_42_ not only exhibits neurotoxicity but also induces mature fibril formation as the hallmark of AD [16,17]. Thus, halting the formation of Aβ_42_ oligomers and subsequent fibril formation is the key to retarding AD progression.

Many natural products, such as polyphenols and flavonoids, exhibit alleviating effects against AD [18,19]. While the exact mechanisms of their function remain unknown, researchers are trying to link the dogmatic antioxidative property of these molecules to their neuroprotective effect [20,21]. Cy-3G exhibits not only anti-tumor and anti-inflammatory activities [22,23] but also neuroprotective effects in cells and rats [24,25,26]. Recently, Liu et al. showed that Cy-3G reduced the amount of intracellular reactive oxygen species induced by Aβ_40_ fibrillogenesis [27]. Furthermore, Cy-3G was found to prevent Aβ_40_ fibrillogenesis and disintegrate its pre-formed fibrils [27]. While Cy-3G is shown to pass the blood–brain barrier and rapidly distribute in the brain, whether Cy-3G could inhibit fibrillogenesis of the more pathologically relevant Aβ_42_ peptide remains unknown [28]. Besides the fact that Cy-3G interacts with the preformed fibril with hydrophobic and electrostatic interactions, the chemical process that drives the disassembly of Aβ_40_, or the more pathologically relevant Aβ_42_ fibril, remains unclear.

Here, based on the experimental evidence that Cy-3G inhibits both the Aβ fibrillogenesis and interacts with the Aβ fibril, we studied the detailed interaction between monomeric and fibrillar Aβ with Cy-3G in silico. The analysis of Cy-3G interacting with different Aβ_42_ polymorphs revealed the chemical driving force of Cy-3G induced Aβ fibril disassembly.

## 2. Materials and Methods

### 2.1. Molecule Preparation

The Cy-3G structure was generated with ChemDraw Pro. 18.0 (CambridgeSoft, Cambridge, MA, USA). AD-relevant solid-state NMR structure of Aβ_42_ (PDB ID: 2NAO) was used to simulate Cy-3G binding to fibrillar Aβ [29].

For monomeric Aβ, one peptide of the above fibrillar structure was derived with PyMol (Schrödinger, New York, NY, USA) and subjected to protein preparation with the BIOVIA Discovery Studio software V16.1.0. The monomeric Aβ was prepared with CHARMm minimization and protonated with a dielectric constant of 10. The pH was set to 7.5, and the ionic strength was set to 0.08. In the following solvation step, 2593 water molecules, 7 sodium and 4 chloride ions were added to neutralize monomeric Aβ using the explicit periodic boundary water model in an orthorhombic cube with a radius of 20 Å and a minimum distance from a boundary of 7.0 Å. The monomeric Aβ was obtained by simulating the standard dynamics cascade with two energy minimization steps. Firstly, 1000 steps of steepest descent minimization and 2000 steps of conjugate gradient minimization, followed by steps of heating, equilibration and production. The monomeric Aβ system was heated from 50 K to 310.15 K in 4 ps with a time step of 2 fs, and equilibrated at 310.15 K for 20 ps with a time step of 2 fs. The SHAKE constraint on hydrogen atoms is applied. Finally, the production step was run at 310.15 K for 200 ps with a time step of 2 ps typed NPT [30].

### 2.2. Molecule Interaction

The molecular interaction between Cy-3G and monomeric, fibrillar Aβ polymorph was performed with the DS CDOCKER module. Random ligand conformations were generated from the initial ligand structure through high-temperature molecule dynamics at 1000 K. The random ligand conformations were refined by grid-based simulated annealing with 2000 steps heating to 700 K and 5000 steps cooling to 300 K and force field minimization. The top 10 poses were saved for subsequent analysis.

### 2.3. Analysis

For Cy-3G to interact with the monomeric and fibrillar Aβ polymorph, the conformation with the lowest energy was selected for molecular interaction analysis. LigPlot (EMBL-EBI Groups, Cambridgeshire, UK) and PyMol were used to analyze the electrostatic, hydrophobic interactions and hydrogen bonding, respectively [31].

## 3. Results

### 3.1. Simulated Single Aβ peptide Solution Structure

**Figure 1:**
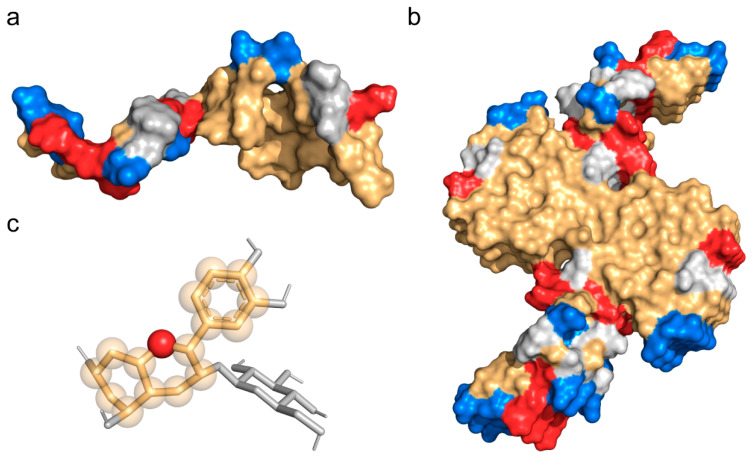
Structure of Cy-3G and different Aβ polymorph. (**a**) Space-filling model of a simulated, single Aβ peptide solution structure. Positive and negative charges on the proteins are shown in tv_red and marine, respectively. The hydrophobic part is shown in light orange. (**b**) Space-filling model of a disease-relevant Aβ fibril structure. (**c**) Space-filling model of Cy-3G showing the positive charge (tv_red) and the hydrophobic character (light orange) on the 2-phenylbenzopyrylium core of this molecule.

In 2020, Cy-3G and amentoflavone were sequentially reported disassembling preformed Aβ fibril [27,32]. Our previous microscale thermophoresis experiment indicated that cyanidin-3-*O*-galactoside preferentially binds to the monomeric Aβ than its fibril polymorph. To investigate whether Cy-3G also exhibits preferential binding to monomeric Aβ, we simulated the solution structure of a single Aβ peptide first. The results showed that a single Aβ exhibited a majorly unfolded structure, with a bent turn structure from F19 to I32, consistent with the previous simulation of A21 to A30 truncate structure and NMR results (Figure 1a) [33,34]. The solvent accessible surface area (SASA) of the hydrophobic region in the 6-peptide Aβ fibrillar polymorph is 4835.67Å^2^, while in one Aβ peptide is 2318.76 Å^2^. The monomeric Aβ peptide alone exposed more hydrophobic surface area in solution than its aggregated fibrillar polymorph.

There is a negative charge on the bulk hydrophobic region of the single Aβ peptide from the deprotonation of E22 and D23 (Figure 1a). Similarly, the fibrillar Aβ structure exhibits large proportions of hydrophobic core regions with negative and positive charges on its sides (Figure 1b). On the contrary, the molecular structure of Cy-3G is a hydrophobic core carrying a positive flavylium cation (Figure 1c).

### 3.2. Interaction between Aβ Fibril and Cy-3G

**Figure 2:**
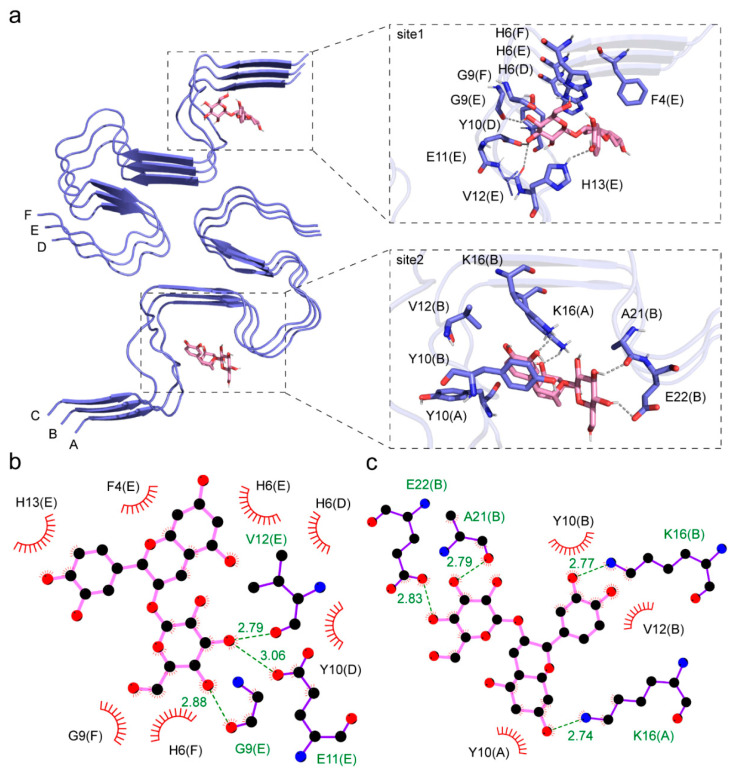
The interaction between Cy-3G and the Aβ fibril. (**a**) The two binding sites between Cy-3G and a disease-relevant Aβ fibril. The cartoon representation is the Aβ fibril structure, and the pink stick presentation is the Cy-3G molecule. The first binding site is the hydrophobic core of one fibril stack, and the other binding site is on the opposite side of the hydrophobic site. (**b**,**c**) are the interaction details between Cy-3G and the first and second binding sites. The dashed lines represent hydrogen bonding, and amino acid residues involved in hydrophobic interactions were shown. For example, F4(E) indicates the fourth phenylalanine in Chain E of the Aβ fibril.

Interestingly, the CDOCK of Cy-3G to the fibrillar Aβ polymorph revealed two different binding sites (Figure 2a). As defined previously, one binding site is at the hydrophobic core of one Aβ stack composed of peptide D-F in the fibril [29]. In the first binding site, Cy-3G formed hydrogen bonds with G9, E11, V12, and H13 in peptide chain E, and hydrophobic interactions with G9, E11, H13, F4, and H6 in peptide chain E, H6 and Y10 in chain D, and H6 and G9 in chain F (Figure 2b). The CDOCKER interaction energy between Cy-3G and this site is −181.31 kJ/mol (Table 1).

The other binding site is at the opposite side of the hydrophobic core in the other Aβ stack of the fibril composed of peptide A-C. Here, Cy-3G formed a hydrogen bond with K16 in peptide chain A, K16, A21, and E22 in peptide chain B, and hydrophobic interactions with K16, Y10 in peptide chain A, K16, A21, E22, Y10, and V12 in peptide chain B (Figure 2c). The CDOCKER interaction energy between Cy-3G and this site is −197.72 kJ/mol (Table 1). 

### 3.3. Interaction between a Single Aβ Peptide and Cy-3G

**Figure 3:**
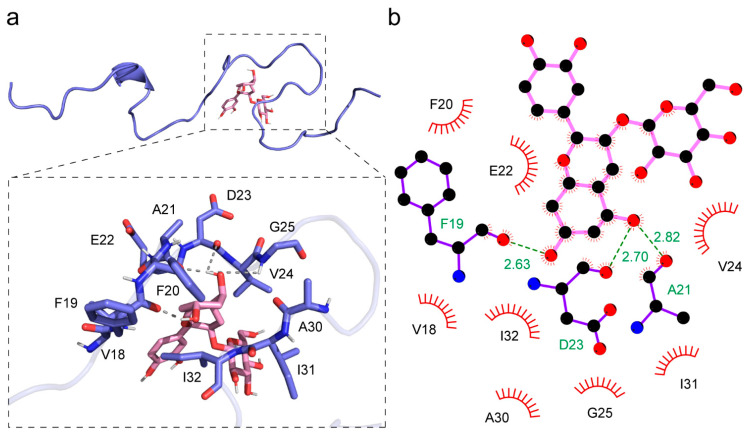
Detailed interaction between Cy-3G and an Aβ peptide. (**a**) The binding site between Cy-3G and an Aβ peptide. The slate carton representation is the Aβ peptide solution structure, and the pink stick presents Cy-3G. (**b**) The interaction details between Cy-3G and the Aβ peptide. The dashed lines represent hydrogen bonding, and amino acid residues involved in hydrophobic interactions were shown.

When Cy-3G binds to a single Aβ peptide, there is only one binding site at the major hydrophobic region (Figure 3a). Here, Cy-3G forms hydrogen bonds with F19, A21, D23, and G25, and hydrophobic interactions with F19, A21, D23, G25, V18, F20, E22, V24, A30, I31, and I32 (Figure 3b). The CDOCKER interaction energy between the Cy-3G and a single Aβ peptide is −43.29 kJ/mol (Table 1).

### 3.4. Preferential Binding of Cy-3G to a Single Aβ Peptide over the Fibrillar Polymorph

It is observed that the monomeric Aβ peptide solution structure exhibits a larger SASA of hydrophobic area. This character draws the interaction between the similarly hydrophobic core of Cy-3G and different Aβ polymorphs. Another significant molecular characteristic between Cy-3G and both Aβ polymorphs is the electric charge they carry. Cy-3G carries a positive flavylium cation on its 2-phenylbenzopyrylium hydrophobic core, and it is this positive charge that drives this small molecule to bind to the region of each Aβ polymorph with negative charges. The detailed consequences of this charge–charge interaction are not discussed here due to limited computational work.

Clearly, Cy-3G interacts with a single Aβ peptide more intensively than the fibrillar polymorph. There are four hydrogen bonds between Cy-3G and Aβ fibril at each binding site, while there are also four hydrogen bonds between Cy-3G and the single Aβ peptide (Table 1). Considering there are six Aβ peptides in one fibril structure studied here, there are more hydrogen bonds formed between Cy-3G and a single Aβ peptide than its fibril polymorph.

In contrast, 11 amino acid residues are involved in the hydrophobic interactions between Cy-3G and a single Aβ peptide. At the same time, the number decreased to 9 when Cy-3G binds to the first site of the Aβ fibril and 7 when Cy-3G binds to the second site (Table 1). There is a much more intensive hydrophobic interaction between Cy-3G and a single Aβ peptide than its fibril polymorph. Thus, Cy-3G preferentially binds to the monomeric single Aβ peptide than the fibril. Aβ peptides aggregate into fibrils and fibrils dissociate into a single peptide; in this chemical equilibrium, pathology favors the aggregation side. In the presence of Cy-3G preferential binding to the single Aβ peptide, this chemical equilibrium could be driven to the side of disassembling of Aβ fibril into its single peptide polymorph. Specifically, the binding of Cy-3G to the F19 and the I32 turn structure fulfills the prediction of a drug candidate targetting this region [34]. Our research only demonstrates a thermodynamic phenomenon, while future stoichiometry studies are needed for the pharmaceutical application of this compound.

This study differs from previous simulations performed on the trimer of Aβ40 and was performed with two different Aβ42 structures: the monomeric, free in solution form, and the hexameric, fibrillar form [27]. As Aβ42 is more AD-relevant than Aβ40, the Aβ fibrillar structure employed in this study is also more relevant to the disease [29]. Here, we found that Cy-3G disrupts the core structure of Aβ fibril, as indicated in the previous study [27], and illustrated its preferential binding to the monomeric Aβ that offers a thermodynamic explanation of this phenomenon.

## 4. Conclusions

The positive charge of the flavylium cation on Cy-3G draws the interaction between this molecule and the negative charges on different Aβ polymorphs. The simulated Aβ single-peptide polymorph displays a larger hydrophobic SASA than its fibril structure, enabling a more intensive interaction between Cy-3G and the hydrophobic region. Thus, the Cy-3G preferentially binds to the single Aβ peptide than its fibril polymorph. The Aβ aggregation/dissociation equilibrium exists between its single peptide and fibril polymorph. It could be the preferential binding of Cy-3G to the single peptide that drives the equilibrium to the dissociation direction, which eventually leads to the disassembling of Aβ fibril.

## Figures and Tables

**Table 1:** Binding parameters of Cy-3G to different Aβ polymorphs.

Aβ Polymorph	CDOCKER Interaction Energy (kJ/mol)	Number of Hydrogen Bonds	Number of Amino Acid Residues Involved in Hydrophobic Interactions
Single peptide	−43.29	4	11
Fibril site1	−181.31	4	9
Fibril site2	−197.72	4	7

## Data Availability

Data is contained within the article.
